# Bridging the Gap for Children With Compound Health Challenges: An Intervention Protocol

**DOI:** 10.3389/fped.2021.721926

**Published:** 2021-12-22

**Authors:** Irene Elgen, Torhild Heggestad, Rune Tronstad, Gottfried Greve

**Affiliations:** ^1^Division of Psychiatry, Department of Child and Adolescent, Haukeland University Hospital, Bergen, Norway; ^2^Department of Clinical Medicine, University of Bergen, Bergen, Norway; ^3^Department of Research & Development, Haukeland University Hospital, Bergen, Norway; ^4^Department of Pediatrics, Haukeland University Hospital, Bergen, Norway; ^5^Department of Clinical Science, University of Bergen, Bergen, Norway; ^6^Department of Heart Disease, Haukeland University Hospital, Bergen, Norway

**Keywords:** health, child, compound condition, health services, intervention

## Abstract

**Background:** During the last decades, there is a major shift in the panorama of diseases in children and adolescents. More children are referred to the specialized health care services due to less specific symptoms and more complex health challenges. These children are particularly difficult to care for in a “single-disease” oriented system. Our objective was to develop an alternative and more holistic approach better tailored to the complex needs of these children.

**Method:** The target patient population is children between 6 and 13 years with three or more referrals including both the pediatric department and the mental health services. Furthermore, to be included in the project, the child's actual complaints needed to be clinically considered as an unclear or compound condition in need of an alternative approach. This paper describes the process of developing an intervention where a complementary professional team meets the patient and his/her family altogether for 2.5 h. The consultation focus on clarifying the complex symptomatology and on problem solving. The bio-psycho-social model is applied, emphasizing the patient's story as told on the whiteboard. In the dynamic processes of development, piloting, evaluating, and adjusting the components, feed-back from the patients, their families, professional team members, and external team coaches is important.

The professional teams include pediatricians, psychologists and physiotherapists. Achieving the transformation from a logistic oriented team where members act separately toward a real complementary team, seems to be a success factor.

**Discussion:** Composing multi-disciplinary and complementary teams was an essential part of the re-designed intervention. Team interaction transforming the professionals from working as a logistic team to act as a complementary team, was one of the important requirements in the process. When re-designing the specialist health service, it is mandatory to anchor all changes among employees as well as the hospital leadership. In addition, it is important to include patient experiences in the process of improvement. Evaluation of long-term outcomes is needed to investigate possible benefits from the new intervention.

**Trial Registration:** Transitioning Young Patients' Health Care Trajectories, NCT04652154. Registered December 3rd, 2020–Retrospectively registered, https://clinicaltrials.gov/ct2/show/NCT04652154?term=NCT04652154&draw=2&rank=1.

## Background

During the last decades, there has been a major shift in the panorama of diseases in children and adolescents (1). Today better diagnostics and improved treatments have allowed more children with previously life-threatening diseases to survive into adulthood. At the same time increasing sub-specialization of medical care is required (2). Concurrently, a growing number of children are referred to the specialized health care services due to less specific symptoms and more complex health challenges (3). Many of these children experience poor quality of life, impaired school attendance, and may end up unemployed as adults (4).

These shifts are challenging the specialized health services and their “single-disease” organization. Too many children with complex or unclear conditions receive un-coordinated services from different medical disciplines and units within the health care system. Patients “crossing over” between somatic and mental health care seem to be particularly affected. Many of these children end up with multiple referrals to the specialized health services (5, 6). Multiple referrals are a conundrum with high risk of unsatisfactory and delayed diagnosis and treatment. To improve the care offered these children, it is necessary to redesign the organization of services and develop new interventions with a more holistic approach. So far, no standardized guidelines have been developed for the care of these children.

To explore the patterns of specialized care for this patient group, we performed a register study in 2016, concentrating on repeated referrals as suggested by the WHO-definition of complex care (6–8). Our findings demonstrated that over a third of young patients had repeated referrals, and nearly 10 percent were referred to both somatic and mental health care during the observation period of 3 years. In addition, when considering non-specific diagnoses as an indicator of unresolved and unclear conditions, we found that a third of the patients still had a non-specific main diagnosis at the end of their hospital contact. Typically, specialists from different medical disciplines meet children with numerous symptoms from different organ systems, consecutively. Each specialist tends to make independent medical decisions. Often the symptoms do not merit for a diagnosis, and because of the unspecific nature of the symptoms, the specialists tend to focus their assessment on the exclusion of specific diagnoses (7). This makes joint and coordinated decisions and problem solving for patients with multiple or ill-defined symptoms difficult. Complementary skills and competence are warranted (9).

To prepare for the development of a more holistic intervention model, families of children with multiple referrals to our hospital were asked about their experiences and expectations of the health care providers (10). Similarly, we asked the family doctors and hospital professionals about their experiences in assessing this patient group. To improve the specialist health care service, it was considered important to focus not only on the logistics, but to create an approach including the perspective of clarifying the condition and problem solving.

**Figure 1:**
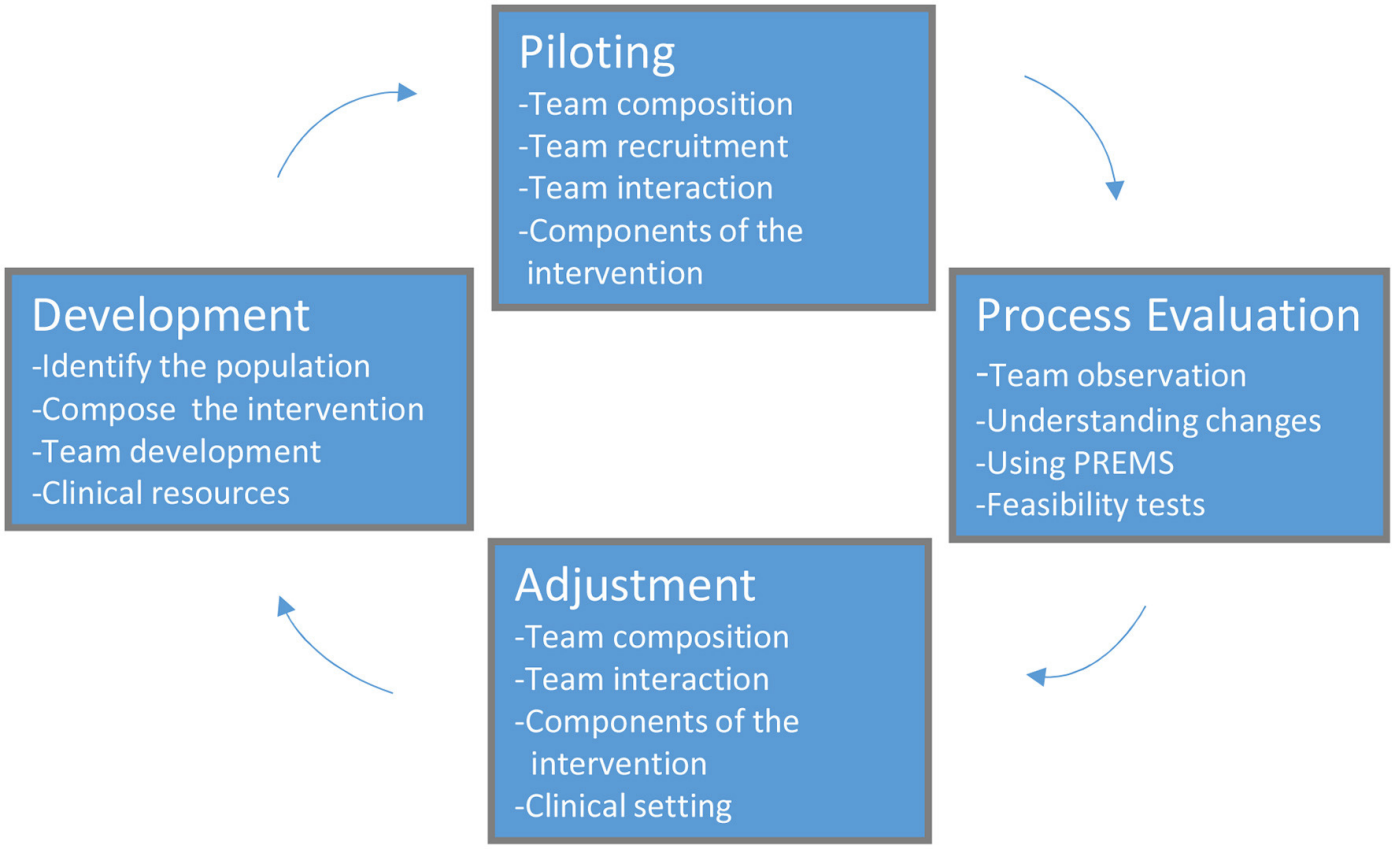
The process of developing and adjusting the clinical intervention.

To our knowledge, no previous study re-designing specialist health care services for this patient group has been conducted, despite the need. Our objective was to develop an alternative approach better tailored to the complex needs of this patient group in our clinical setting. The Medical Research Council (MRC) framework for evaluating complex interventions was taken as the basis for the present research (11). In Figure 1, we present our modification of the model, including the four major steps in the transformation process: Development, Piloting, Evaluation, and Adjustment, as well as their specific elements.

## Methods/ Design

Key factors in designing an intervention model of this kind are target population, components of the intervention, professional team composition and interaction, as well as process and feasibility measures.

### Population and Inclusion Criteria

As reported in a preceding hospital register study of children between 6 and 13 years, more than a third of patients had repeated referrals, and 9% of all patients were referred to both the pediatric department and the child and adolescent mental health services (CAMHS) (6). The pediatric sub-specialties most often included in such combined referrals were found to be gastroenterology and neurology. In an audit of 250 medical records randomly selected from these patients with multiple and combined referrals, we found that most of them were settled with well-established treatment- and follow-up programs. However, almost 15% of the children had multiple unclear health complaints and cumbersome and non-conclusive care pathways within the hospital (7).

According to the register research (6, 7), the initial four criteria were included in the operationalization of inclusion criteria:

1. Children aged 6 to 13 years referred to specialist health care services (Haukeland university hospital).
2. Three or more previous referrals within the last three years where at least one referral to CAMHS and one to the pediatric department.
3. The different referrals should all be independent of each other, and the most recent referral should be to ambulatory care.
4. The current referral further needed to be medically evaluated whether the actual health complaint(s) could be considered as an unclear or compound condition and in need of an alternative intervention. This evaluation was performed based on information from the referral letter and the patient's medical record/history by a senior clinician (IBE). Typically, selected patient histories were characterized by repeated referrals without any diagnostic conclusion or with unsuccessful treatment resulting in increased functional loss like school absence.

Parents of selected children meeting the inclusion criteria were invited by a phone call from a research nurse and given written information regarding the study. They were informed that the hospital had received a referral from the family doctor to a pediatrician; however, the medical record indicated need of a more interdisciplinary approach. The parents could decide whether they wanted to participate in the study or follow standard procedure meeting only the pediatrician. Written consent was obtained on the day of the consultation.

### Team Composition and Interaction

After register studies in 2016–18, we started in 2019 developing the intervention. The project was initiated to improve the medical services offered in our hospital to children with multiple referrals for combined mental and somatic issues. Thus, it was reasonable to include both a pediatrician and a psychologist in the team. In our registry study, children with health problems related to gastrointestinal or neurological systems constituted the largest groups (6, 7). Thus, colleagues with long experience in gastroenterology and neurology were invited to participate in the professional teams. From the reviews of 250 medical records, it was perceived that many of the tangible patients ended up seeing a physiotherapist (7). Thus, physiotherapists with long experience in testing children both with somatic and mental health problems were invited to join the teams. The intention was that the teams should work to take mutual advantage of their professional background as pediatricians, psychologists, and physiotherapists.

In the hospital there is a long tradition for collaboration between different professions and professionals from different fields e.g., in the surgical theaters and trauma teams. However, most well-functioning hospital teams generally have highly defined roles and responsibilities (12). In the new chosen model of intervention, no such pre-defined roles were established.

The intention was to develop *complementary teams* where the team members use each other as “diagnostic tools” both in understanding what the patients and their families communicate as well as observing them. We judged it almost impossible for the professional members of the teams to reach this goal independently. Even more so if they should be able to include patient and parents in the team to reach a consensus on how to understand the patient's condition i.e., shared decision-making regarding both assessment and treatment of the child. Therefore, it was decided to engage an experienced external team coach. Simultaneously, the team development was included in a team research project using participating observational methods.

In the process of piloting the teamwork, the coach organized individual sessions and day seminars with the team members, and furthermore, observed all teams in action during the interventions. Different aspects of teamwork were addressed, focusing on team function and team tasks as illustrated in Table 1.

**Table 1:** Alternative outcomes of teamwork: Green cells illustrate better outcome than if the members work individually, orange means equal outcome as if the team members work individually, and red means worse outcome than if the team members work individually.

		**Well-defined team task**	
		**Yes**	**No**
Well-functioning team	Yes		
	No		

The coach challenged the team members on how to define the team task for each patient/clinical setting. Usually, the team task is well-defined, like in trauma teams. However, in the present setting the teams needed to define each time what tasks could best be approached together and what to approach individually. No cases were alike, and it often ended with a mix of joint and individual assessments. The team members found the variability difficult, but the awareness of this challenge really speeded up the team development resulting in almost a transformation of the teams.

A prerequisite for the intended team development was more stability in the teams, i.e., less swapping of members between teams and that they worked together more frequently. First, we restricted the possibility to move between teams. Such movements resulted not in four teams as planned, but rather 9 or 10 different team compositions. Secondly, we invited a second psychologist to join the project. Today, we have four different teams, two focusing on gastroenterology and two on neurology. Four pediatricians, three physiotherapists and two psychologists are engaged in the teams.

It might appear easy, but it is not, to use each other as *diagnostic tools* in reaching a joint perception of the patient and his/hers family. Most professionals are educated to make individual assessments and decisions but are far less trained in using other team member's different backgrounds as complementary instruments. Despite the professional part of the teams had a “flat” hierarchical structure with all professional team members as equal participants, it soon became obvious that one team member had to orchestrate the intervention to ensure that the program with the different components was followed and completed. It was necessary to choose the team leader before the intervention started. We have also experienced that how the different participants are seated in the room, who starts the conversation, and the use of tools like whiteboard, are important factors for the outcome of the intervention.

To summarize, team composition and interaction transforming the professionals from working as a logistic team to act as a *complementary team*, is one of the important requirements in this new intervention.

### Components of the Intervention

Based on the Bio-psycho-social model and the guidelines from Kozlowska and Turn the intervention took form. Kozlowska has outlined a framework for interviewing families whose children present with medically unexplained symptoms (13). This model aims to connect body, mind and social environment in understanding the symptom presented in the child (14). Factors maintaining the symptoms should be explored as well as components of stress. Further, self-regulation is another issue that should be addressed in order to promote health to the child and his/her family.

Based on our traditional thinking, the first draft of intervention was a well-organized setup of three consecutive consultations with the three professionals individually. Afterwards the professional team members had a secluded discussion of their findings before they presented a conclusion and a plan to the patient and the family (Table 2). However, already after a few tests, this setup seemed too stressful and time consuming for the families. Families also expressed dissatisfaction with having to repeat their history and the child's health problems three times to the different professional team members.

**Table 2:** The initial draft for the components of the intervention.

**Time schedule**	**Patient**
20 min	Introduction
	Meets the whole team
10 min	Short discussion in the team without patient
45 min	Meets the pediatrician
45 min	Meets the physiotherapist
45 min	Meets the psychologist
15 min	Short discussion in the team without patient
15 min	Whole team, summary, conclusion and further plan
4 weeks later	Follow up
45 min	Whole team

According to the experiences disclosed above, a revised schedule for the intervention was set up (Table 3). Before the intervention starts, the patient and her/his family answer questionnaires regarding health status and previous experiences with the health care systems. Concurrently, the professional team prepares together for the intervention based on information obtained from the medical record and referral letter, and decide who should be orchestrating the intervention. The intervention starts with patient and parents sharing their concerns and health complaints to the joint team. Then the professional members of the team discuss secluded and customize a program for the rest of the intervention emphasizing on what further information is needed and what subjects to assess. Then the assessment takes place. This part is individualized to each patient and could include further interviews and/or clinical examination. Given the heterogeneous nature of this patient group, no standardized approach is given, but must be individualized according to the situation presented. Before rounding up, the professional part of the team summarizes the assessment secluded. Finally, the complete team including patient and his/her parents make a shared decision on how to understand the condition, if further clinical examinations are needed, and establish a treatment plan.

**Table 3:** Final structure for the intervention.

**Time schedule**	**Patient and family**	**Professional team**
30 min pre-intervention	Meet the research nurse and fill out the questionnaires	Prepare the intervention
15–30 min	Patient and its family share their concerns and health complaints to the joint team
15 min		Secluded planning of the intervention
45–60 min	Intervention/ Diagnostic assessment
15 min		Secluded reflection on findings
	Summarizing the day and agreement on further interventions.
4–6 weeks later	Follow-up

As requested by family doctors, it has been emphasized that the assessment should conclude with one joint statement from the professional team, where the pediatrician, physiotherapist, and psychologist reach a consensus. Further, the team was encouraged to include the patient and his/ her family in a common understanding of the problem and the proposed treatment- or follow-up plan.

#### Narrative Diagnostics

Aiming to support the child with life-coping strategies one has to take the perspective of the child regarding communication. It is too easy to address the child by many questions regarding symptoms. Addressing the symptom of the child through externalizing children's “problem” using play or narrative elements is described as an alternative tool when children are unable to verbalize their inner ailment (15). Grant and Ushers (16) highlight using whiteboard as a tool that not only slow down the conversation, but also help the professionals to listen and humbly take part in the child's world and understanding of their situation. Instead of words, the child can draw a picture expressing themselves in a different way. Initially in our pilot testing the intervention, this method was tried out by the teams. Feed-back from the families supported such a concept as an important tool in communication with the child, and furthermore, that the child experience that they are listened to.

#### Treatment Plan

After listening to the story of the patient and his/her parents and understanding the child's health challenges, it must be decided if further medical evaluations are necessary in order to fully understand the child's s health problems. Agreeing on and creating a treatment plan is the final step of the consultation. After the first 10 pilot tests, it became obvious that most families wanted to make a new appointment with the team after 4 to 6 weeks. Meanwhile, eventually further assessment or psychological support from team members could be accomplished.

#### Clinical Setting

Our project acquired resources from the national research council to perform the register studies (6, 7). However, it has been more difficult to get external support to develop and test the new intervention. Accordingly, we have chosen to implement the pilot in the ordinary clinical setting. To make this possible, it was necessary to include the leaders of the involved departments in our planning as the project's steering committee. Their acceptance and support have been mandatory to be able to prioritize the involvement of the team members. In addition, the core researchers have used a lot of time on information regarding the objectives of the new intervention as well as our experiences in composing teamwork.

### Process and Feasibility Measures

#### The Process

The process of developing an alternative and innovative intervention model, recognizing the need for a structured and holistic clinical outpatient approach, has been long and challenging. Understanding and recognizing the need for changing attitudes and ways of cooperation has been essential. There has been a continuous communication with the team members through regular lunches for the professional team members and core research group where different aspects of the project have been addressed. Midway we organized a workshop focusing on feedback from the users, coach, experiences from the team members, and the researcher following the teams. The final intervention model as described was anchored at the steering committee.

#### Feasibility Measures

The project has developed a set of feasibility outcome measures. User's experiences are mandatory in the evaluation and further improvement of the provided health care. In the present study, three users are identified: Patient (child), parents, and professionals. Questionnaires targeting the different users have been developed for the present study.

In order to get information from *the children* themselves on how they experience meeting a complementary team of different professionals, they are asked to answer a five items questionnaire; The consultations and relational empathy (CARE) measures are given in Table 4 (q 17–20) (18–21).

**Table 4:** Areas covered in the different questionnaires regarding feasibility of the new intervention.

**Responder**	**Questions**
Patient	How content are you with the help you received today?
	1 = Not at all to 4 = Very content
	If a friend needed help like what you have received today would you recommend this intervention:
	1 = Not at all to 4 = Yes
Patient	How was the team at making you feel happy and relaxed?
CARE^*^	*(Being friendly and caring and making you feel calm)*
	1 = Not very good to 5 = Excellent
	How was the team at asking questions and letting you talk?
	*(Being interested in you and giving you time to speak)*
	1 = Not very good to 5 = Excellent
	How was the team at listening and understanding?
	*(Paying attention and knowing the things you find difficult)*
	1 = Not very good to 5 = Excellent
	How was the team at explaining things?
	*(Answering questions, giving you a clear information and instructions)*
	1 = Not very good to 5 = Excellent
	How was the team at making a plan?
	*(Encouraging you, talking about what to do next, involving you as much as you want)*
	1 = Not very good to 5 = Excellent
Parents	How content are you with meeting the team?
	1 = Not at all to 4 = Very content
	If a friend needed help like you have received today would you recommend it?
	1 = Not at all to 4 = Yes
Team	Was the intervention today useful for the child?
	1 = Not at all to 4 = Very useful
	Was the intervention today useful for the parents?
	1 = Not at all to 4 = Very useful

* *CARE: The consultations and relational empathy questionnaire, Mercer S*.

Prior to the intervention, *the parents* will answer a questionnaire regarding their previous experiences with the specialist health services. After the intervention, they answer a questionnaire regarding their opinion/experience with the intervention and the professional team (Table 4). After each intervention, the *professionals in the team* are evaluating themselves by answering a questionnaire together. Areas addressed are how the team perceived the complexity of the patient's condition, as well as giving their evaluation of how useful the team anticipated this intervention was for the child and the family (Table 4).

## Discussion

Based on our clinical experiences, the demonstrated complex patterns of care for patients with repeated referrals, as well as the experiences of families, we aimed to develop and pilot a new intervention. The intervention include multidisciplinary, complementary teams consisting of pediatricians, psychologists, and physiotherapists. In the process, a stepwise progression was applied using repetitive evaluations and adjustments was followed as described in Figure 1 (11).

### Population—Inclusion Criteria

Children with multiple and unclear health complaints and, therefore, multiple referrals, are well-known in clinical practice. However, to define and operationalize their characteristics unequivocally is challenging. Our project aims to identify the children who might benefit from this specific innovation of redesigned health care.

Only focusing on multiple referrals are not suitable as a single criterion for complexity even if they include referrals both to somatic and mental health care. Many children have entirely independent health complains like a broken leg, medial otitis, and ADHD, which are better managed separately. However, from our previous registry study, there was a difference in children with less compared to more than three referrals including one to pediatrics and one to CAMHS (6). Therefore, we used more than three referrals including one to pediatrics and one to CAMHS for the primary selection of children. However, the complexity and the unclear symptom picture are important features as well as the functional status like school attendance. Therefore, an experienced clinician making a secondary and final selection based on the referral letter and the child's medical record is recommended.

The professional team members argued already from the beginning for including children with unclear health problems resulting in school refusal without having been referred to CAMHS, and for a higher upper age limit. In their clinical practice, the main problems were related to teenagers, 13–16 years of age. This criterion needs to be tested in future intervention studies. In the registry study (6, 7), children with referrals to either the gastroenterology or the neurology units made up the majority of patients. Thus, pediatricians working within these two units were recruited to the teams. The pediatricians were, however, reluctant to include patients in need of assessment from other medical disciplines. Accordingly, only patients with the latest referral to either gastroenterology of neurology were included in the pilot study. However, there is reason to assume that our intervention is generalizable to other medical disciplines.

### Team Composition and Interaction

The construction of teams consisting of a pediatrician, physiotherapist and psychologist was hitting the target already from the start (6, 7). The teams appear able to deal with patients with a broad specter of complex, unsolved and unspecific health problems. However, there are some prerequisites; i.e., one should pick experienced professionals who still have maintained a relatively broad medical perspective. Thus, it is a great advantage that all the pediatricians in the teams participate in general on-calls in the pediatric department.

Transforming teams from “logistic” interdisciplinary teams to “complementary” teams was more demanding than predicted. All the team members are used to work in interdisciplinary teams where their roles and responsibilities are well-defined. They are also used to discuss patients with colleagues from other professions. It is necessary for the professionals in the teams to know each other well-enough to have confidence in each other professionally, but also in facing the patient and the parents. Trust is built over time and requires stable teams that meet frequently enough not to start over again every time. Thus, we recommend that all teams meet at least every second week, and swapping between teams should be avoided. Another lesson learned is the need of debriefing for the team due to the complexity and often heavily burdened situation for the patient and family. This also contributed to the building of trust within the teams.

Initially, the hierarchical thinking that the physician should lead the conversation prevailed. This always led the intervention in a somatic direction. However, the patient and the family could more readily address other and even non-medical issues when other team members opened and orchestrated the conversation. The importance of this shift was encouraged by the external coach who often commented on the hierarchical culture within the teams, and challenged the professionals to change mindset. Therefore, recommendations from piloting is to let non-medical professional lead the conversation.

### Intervention Components

Meeting a team of three professionals in addition to both parents, could be overwhelming for a child. However, in the test period both children and parents expressed their contentment with the holistic approach and were comfortable in the situation. Having the psychologist in the team was a strength reported from several parents even when they beforehand expressed skepticism toward participation of a psychologist.

In its final setup, each intervention last 2.5 h, where patient, family members and the professionals in the team spend most of the time together. This could seem too long, but after testing the model both professionals and families agreed that spending that long time together, gave the family confidence that the professionals had understood their problems and taken the family seriously. Early it appeared that many patients and their families need a second appointment, sometimes after further examinations or individual appointments with either the physiotherapist or psychologist. Four to six weeks after the first intervention has shown to be the best in most cases. Nevertheless, it is mandatory that the children meet the same team as the first time.

### Process and Outcome Measures

In the processes described here, we have focused on creating the intervention and the feasibility measures. Both feasibility measures as well as short- and long- term outcome measures are needed. So far, children have been able to provide useful information through the CARE questionnaire and we recommended this in future studies.

For effect measures, mental and somatic health status, QoL, PREMs, as well as school attendance and the use of health services following the intervention, are fundamental dimensions that will be covered in our future studies. Further, we plan to include a control group in the last step of a RCT study.

Another aspect of outcome measures are the financial consequences for the health care system as previous mentioned by Heggestad (6). No consensus is given in how to economically evaluate implementation of new interventions (17). However, economic outcomes will be obtained both in terms of use of healthcare services, parent's work ability as well as children's school attendance.

## Conclusion

To develop an alternative approach better tailored to the complex needs of children referred repeatedly to different medical disciplines, turned out to be a demanding process. Composing multi-disciplinary and complementary teams was essential. The interaction within the teams, transforming the professionals from working as a logistic team to act complementary, was a decisive in the process. Furthermore, this transformation seems to be the success-factor. When re-designing the specialist health service, it is mandatory to anchor all changes among employees as well as the hospital leadership. In addition, it is important to include patient experiences in the process of change.

## Data Availability Statement

The original contributions presented in the study are included in the article/supplementary material, further inquiries can be directed to the corresponding author.

## Ethics Statement

The studies involving human participants were reviewed and approved by the Regional Committee for Medical Research Ethics (REK Vest) (REC 2018/344). Written informed consent to participate in this study was provided by the participants' legal guardian/next of kin.

## Author Contributions

IE, TH, RT, and GG have contributed in developing the protocol and writing the manuscript and fulfill the Vancouver criteria for participation. All authors read and approved the final manuscript.

## Conflict of Interest

The authors declare that the research was conducted in the absence of any commercial or financial relationships that could be construed as a potential conflict of interest.

## Publisher's Note

All claims expressed in this article are solely those of the authors and do not necessarily represent those of their affiliated organizations, or those of the publisher, the editors and the reviewers. Any product that may be evaluated in this article, or claim that may be made by its manufacturer, is not guaranteed or endorsed by the publisher.

## Abbreviations

WHO: World health Organization
MRC: Medical Research Council
TpT: Transitioning young patient's health care Trajectories
CAMHS: Child Mental Health Service
CARE: Consultations And Relational Empathy
ADHD: Attention Deficit Hyperactivity Disorder
QoL: Quality of Life
PREM: Patient Reported Experiences Measures
RCT: Randomized Controlled Trial.
